# A pilot evaluation of the artificial intelligence system CAD-EYE to optically characterise lesions in inflammatory bowel disease surveillance

**DOI:** 10.1177/26317745251363517

**Published:** 2025-08-18

**Authors:** Sherman Picardo, Shankar Menon, Kenji So, Kannan Venugopal, Wendy Cheng, Krish Ragunath

**Affiliations:** Division of Gastroenterology and Hepatology, Royal Perth Hospital, Victoria Square, Perth, WA 6000, Australia Curtin University, Perth, WA, Australia; Royal Perth Hospital, Perth, WA, Australia; Royal Perth Hospital, Perth, WA, Australia; Royal Perth Hospital, Perth, WA, Australia; Royal Perth Hospital, Perth, WA, Australia; Royal Perth Hospital, Perth, WA, Australia; Curtin University, Perth, WA, Australia

**Keywords:** cancer, colitis, dysplasia, inflammatory bowel disease, surveillance

## Abstract

**Background:**

Patients with inflammatory bowel disease (IBD) have an increased risk of colorectal cancer. Endoscopic surveillance is recommended but is challenging due to the presence of active inflammation, flat dysplasia and inflammatory pseudopolyposis. CAD-EYE, an artificial intelligence (AI) powered endoscopic module by FUJIFILM, optically characterises lesions in real time. The aim of this study was to evaluate the accuracy of CAD-EYE in IBD surveillance.

**Methods:**

Ninety-seven lesions were identified from 38 patients with IBD, undergoing surveillance at a single centre. Non-magnified, still images of lesions identified during the procedure were captured, followed by characterisation by CAD-EYE as neoplastic or hyperplastic (non-neoplastic) prior to resection. Inflammatory pseudopolyps were imaged and only resected based on the physician’s discretion. Images of lesions identified were characterised by two expert IBD clinicians. The accuracy of CAD-EYE was assessed for all lesions (composite of histology for resected lesions and expert-verified non-resected pseudopolyps). For the resected lesions, the accuracy of CAD-EYE was compared to expert characterisation.

**Results:**

CAD-EYE correctly characterised 92/97 lesions (94.8%) with a sensitivity of 80.0%, specificity of 97.6%, positive predictive value of 85.7% and negative predictive value of 96.4% for neoplastic lesions. For resected lesions, diagnostic accuracy was similar between CAD-EYE (93.0%) and expert characterisation (88.4%), with no statistically significant differences in sensitivity.

**Conclusion:**

CAD-EYE demonstrated its utility in IBD surveillance with excellent accuracy in the characterisation of lesions, including inflammatory pseudopolyps. Larger studies are required to confirm these findings, particularly for flat dysplasia.

## Introduction

Patients with inflammatory bowel disease (IBD) have an increased risk of developing dysplasia and colorectal cancer.^1^ Although the rates of cancer are declining, largely due to the use of more effective therapies to control disease activity, it remains one of the most serious complications of IBD.^2^ The pathogenesis of colorectal cancer (CRC) in patients with IBD follows an inflammation-dysplasia-carcinoma sequence, and therefore, early detection and resection of precancerous lesions can prevent the development of cancer.^3^

Endoscopic surveillance to identify these early changes is an important aspect of IBD management and has been associated with reduced risk of interval cancer as well as a reduction in mortality from CRC.^4^ IBD surveillance is challenging due to the presence of inflammatory pseudopolyps, the presence of active disease and flat dysplasia and advanced endoscopic techniques including dye-spray chromoendoscopy are recommended to increase the detection of abnormal lesions. Several guidelines have been published that provide recommendations on surveillance intervals and standards.^5,6^ Unfortunately, these guidelines are poorly adhered to in clinical practice, due to limited awareness of guidelines as well as a lack of experience with some of the recommended techniques.^7^

Artificial intelligence (AI) based technologies are increasingly being used in gastrointestinal endoscopy.^8^ Several AI-based tools have been developed which can aid clinicians during endoscopy, including improved mucosal visualisation as well as in the detection and characterisation of abnormal lesions.^9^ CAD-EYE is an artificial intelligence-powered endoscopic module developed by FUJIFILM that automatically identifies and marks abnormalities consistent with polyps in real time.^10^ Once a suspected polyp is detected, CAD-EYE can also then characterise the polyp as neoplastic or hyperplastic (non-neoplastic). Our aims were to evaluate CAD-EYE with regards to characterisation of lesions in patients undergoing surveillance procedures for IBD.

## Methods

Thirty-eight patients with inflammatory bowel disease who met criteria for dysplasia surveillance as per Australian guidelines^6^ underwent colonoscopy with CAD-EYE assistance as part of an evaluation of this system, between January and December 2023, at Royal Perth Hospital, a tertiary IBD centre.^10^ Patients were included if they were 18 years or older and had ulcerative colitis extending past the rectum or Crohn’s disease affecting at least 1/3 of the colon, with disease activity at least 8 years after symptom onset, unless they had concurrent primary sclerosing cholangitis.^6^ Patients were excluded if they had active inflammatory bowel disease or inadequate bowel preparation, defined as a Boston bowel preparation score less than 6.^11^ Ninety-seven lesions, imaged with CAD-EYE, from these 38 patients, were obtained from the endoscopy image database for evaluation in this study. This study adhered to the STROBE guidelines (Supplemental File 1).

### Surveillance procedures

All surveillance procedures were performed as per current Australian clinical guidelines.^6^ Linked Colour Imaging (LCI), an image-enhanced endoscopy mode by FUJIFILM, was used during withdrawal.^12^ If a lesion was detected by the operating physician, non-magnified images were captured with blue light imaging mode as well as with CAD-EYE. CAD-EYE automatically characterised the lesion as neoplastic or hyperplastic.

All concerning lesions were either biopsied or resected as per current clinical practice. Inflammatory pseudopolyps, which are often seen in patients with inflammatory bowel disease and have no malignant potential and are generally not resected. Lesions in keeping with inflammatory pseudopolyps as per the treating proceduralist were imaged by CAD-EYE but not resected. These images were included in the study (a maximum of five images of non-resected pseudopolyps per patient) if verified as inflammatory pseudopolyps by two expert IBD physicians (SM and KS). These were included to assess the ability of CAD-EYE to characterise inflammatory pseudopolyps as non-neoplastic. All resected lesions or biopsy specimens were histologically assessed and characterised by an expert gastrointestinal pathologist.

### Image selection

**Figure 1.**
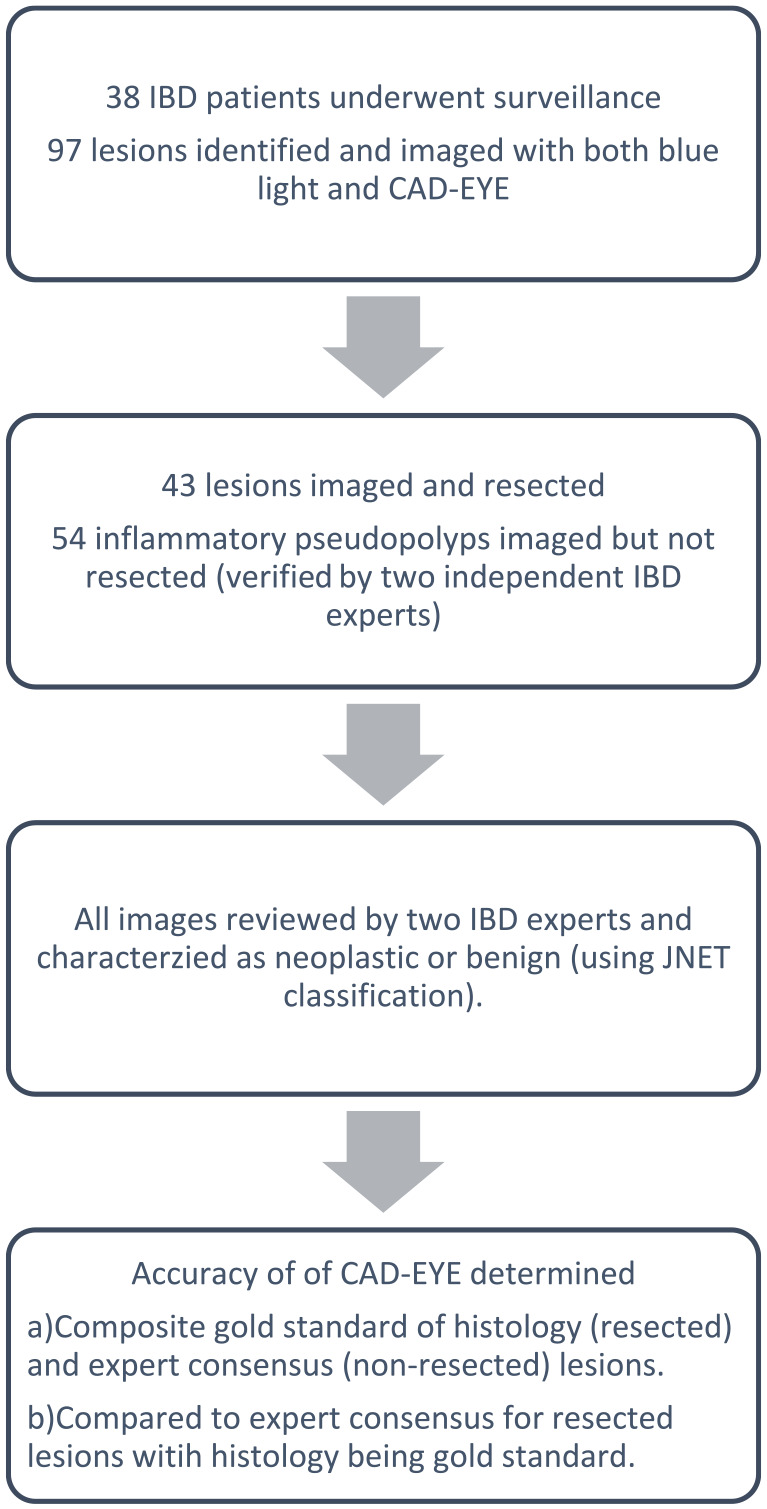
Flow chart of study design.

All pairs of blue light and CAD-EYE images of lesions were collected from an endoscopy image database. The blue light images were reviewed by two expect IBD physicians (SM and KS) and were classified based on appearances (Japan NBI expert team—JNET) classification as benign or neoplastic.^13^ If there was any discordance in classification between reviewers, a third reviewer (SP) provided a consensus diagnosis. A total of 97 images of lesions were included. The study design and methods are summarised in the flowchart (Figure 1).

### Study objectives

The primary objective was the accuracy of CAD-EYE in the detection of neoplastic lesions. CAD-EYE was compared to a gold standard composite measure of lesion characterisation based on histology for the resected lesions and expert verification for the non-resected inflammatory pseudopolyps. As a secondary objective, the accuracy of CAD-EYE was compared to the accuracy of the expert characterisation for resected lesions using histology as the gold standard.

### Statistical analysis

Statistical analyses were performed using Stata Statistical Software: Release 18.^14^ A descriptive analysis was conducted for continuous variables, reported as a median and interquartile range, while categorical or binary variables were presented as proportions or percentages. Sensitivity, specificity, positive predictive value (PPV), negative predictive value (NPV) and accuracy (correct diagnosis) were calculated. McNemar’s exact test was used to compare the sensitivity and specificity of CAD-EYE and expert characterisation for resected lesions. Significance was defined as a *p* value less than 0.05.

### Ethical considerations

Ethics approval was obtained through the Western Australia (WA) Health: Governance, Evidence, Knowledge, Outcomes (GEKO) system for assessment of audit and quality activities (QA50366).

## Results

Ninety-seven lesions were identified and included in the study from 38 patients undergoing surveillance colonoscopy. The baseline characteristics of patients and colonoscopy outcomes are summarised in Table 1.

**Table 1.** Baseline characteristics.

Charactersitic	*n* = 38
Sex (male)	19 (50%)
Age (years)	52 (44–60)
Disease type	
Crohn’s disease	18 (47%)
Ulcerative colitis	20 (53%)
Total procedure time	27 (21–36)
Withdrawal time	19 (15–28)
Dysplasia detection rate	10/38 (26%)

# Categorical variables presented as number (%) and continuous variables presented as median (interquartile range).

Of the 97 lesions identified, 43 were resected with histopathology demonstrating 22 hyperplastic lesions, 11 adenomas, 4 dysplastic sessile serrated lesions, 4 inflammatory pseudopolyps and 2 lymphoid tissue. There were no cancers identified. Three of these lesions were flat, of which one was dysplastic. The remaining 54 lesions were inflammatory pseudopolyps that were imaged but not resected.

**Figure 2.**
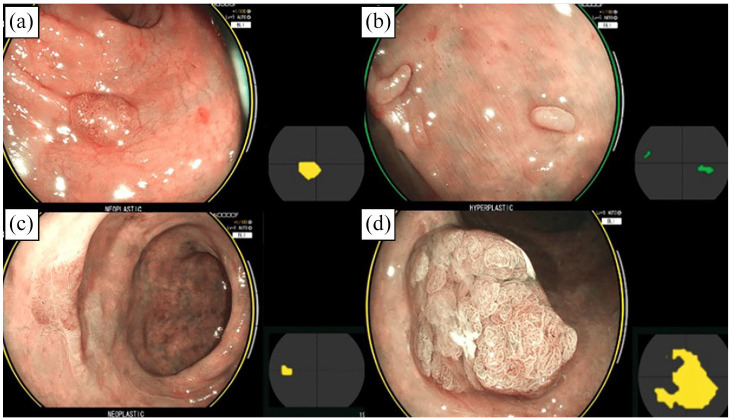
CAD-EYE correctly characterises (a) sessile adenoma (neoplastic), (b) inflammatory pseudopolyp (hyperplastic), (c) flat adenoma (neoplastic) and (d) sub-pedunculated adenoma (neoplastic).

CAD-EYE correctly characterised 92/97 lesions (94.8%) with a sensitivity of 80.0%, specificity of 97.6%, PPV of 85.7% and NPV of 96.4% for neoplastic lesions (Table 2). Some examples of CAD-EYE characterising lesions are demonstrated in Figure 2.

**Table 2.** Accuracy of CAD-EYE.

	Lesion diagnosis*		
	*n* = 97	Neoplastic	Non-neoplastic
CAD-EYE	Neoplastic	12	2
	Non-neoplastic	3	80

* Composite gold standard diagnosis based on histology for resected lesions and expert verification for inflammatory pseudopolyps.

For the 43 resected lesions, CAD-EYE correctly characterised 40 lesions (accuracy 93.0%, sensitivity 80.0%, specificity 100%, PPV 100%, NPV 90.3%). This was numerically superior to expert characterisation 38/43 (accuracy 88.4%, sensitivity 66.7%, specificity 100%, PPV 100%, NPV 84.9%). However, the differences in sensitivity and specificity were not statistically significant (*p* = 0.50).

CAD-EYE correctly characterised 52/54 (96.3%) non-resected pseudopolyps as being hyperplastic (non-neoplastic).

## Discussion

This is the first reported study that evaluates the diagnostic performance of CAD-EYE, an artificial intelligence system, in characterisation of lesions in IBD surveillance. It demonstrates the utility of using this system to aid physicians as well as outlines some of its limitations. AI systems are increasingly being used in a number of applications in IBD management, including endoscopic evaluation of disease activity, histologic interpretation, as well predicting treatment responses to therapy.^15^ There however has been limited evaluation of AI systems in the setting of dysplasia surveillance in IBD.

CAD-EYE is one of the first computer-aided detection systems to combine both the detection of lesions as well as characterisation using the same platform.^10^ Other AI systems, such as GI Genius and EndoBrain, have shown similar capabilities in polyp detection and characterisation; however, CAD-EYE’s integration of detection and characterisation in one platform is a notable advancement. A European study evaluated CAD-EYE in a general population and found that CAD-EYE was able to identify polyps before endoscopists in 32% of cases.^16^ The diagnostic accuracy of the CAD-EYE system was similar to that of the endoscopists. A more recent large multicentre randomised controlled trial of population screening colonoscopy in the United States demonstrated that the CAD-EYE system led to significantly higher adenoma per colonoscopy compared to conventional colonoscopy (0.99 ± 1.6 vs 0.85 ± 1.5, *p* = 0.02). There were, however, no significant differences in the adenoma detection rate.^17^

IBD surveillance is more challenging than population-based screening colonoscopy due to inflammatory pseudopolyps, which can mimic or conceal dysplastic lesions, the presence of active disease, which makes lesion detection difficult, as well as the presence of flat dysplasia.^7,18^ Our study focussed on characterisation of lesions and demonstrated an excellent overall accuracy of 94.8% with a high positive and NPV of 85.7% and 96.4% respectively, in the detection of neoplastic lesions. In addition, CAD-EYE was found to have a high accuracy of 96.3% in correctly characterising inflammatory pseudopolyps. The diagnostic accuracy for resected lesions was 93%, slightly higher than expert characterisation at 88.4%, but the differences were not statistically significant. CAD-EYE’s high accuracy suggests it can significantly aid endoscopists in real-time decision making during IBD surveillance. It may assist endoscopists in providing real-time characterisation to support or guide decisions to resect or biopsy a lesion. It may also reduce the need for non-neoplastic lesion resections, shorten overall procedure times as well as reduce costs. A dedicated randomised controlled trial of CAD-EYE would be useful in evaluating these outcomes.

There were several limitations with our study. The main limitation was the small sample size of dysplastic lesions and the limited diversity of lesions (particularly flat dysplasia) included in the study. This was a pilot study, and a formal power analysis was not performed. Flat or non-conventional dysplastic lesions are often seen in patients with IBD and are the main reason regular surveillance and use of chromoendoscopy are recommended.^19^ Larger cohorts with more diverse lesions are needed to test the CAD-EYE system to detect and characterise flat dysplasia.

A second limitation was that magnification was not used for image capture and lesion characterisation. Magnification has been demonstrated to improve the observation of the mucosal crypt pattern and aid in distinguishing between neoplastic and non-neoplastic lesions.^20^ Use of magnification may have improved the accuracy of lesion characterisation among the IBD experts. Future studies should consider incorporating magnification techniques.

Another limitation was that the study was conducted at a single centre, which may limit the generalisability of the results. CAD-EYE may also potentially reduce costs associated with IBD dysplasia surveillance; however, it was not a focus of our study. Future research should evaluate the economic impact of implementing CAD-EYE in clinical practice.

The CAD-EYE system is a great AI tool for the detection and diagnosis of lesions during colonoscopy. At present, it is only able to characterise lesions as neoplastic or hyperplastic (non-neoplastic). It would be useful for further iterations of this system to classify lesions based on subtype (adenoma, sessile serrated lesion, inflammatory pseudopolyp, hyperplastic polyp) and provide a grade of dysplasia.

## Conclusion

This pilot study highlights the impressive potential of CAD-EYE in lesion characterisation during IBD surveillance. CAD-EYE demonstrated an excellent overall accuracy of 94.8%, effectively distinguishing neoplastic from non-neoplastic lesions in real time. It was comparable to expert IBD clinician characterisation for resected lesions and was also accurate in characterisation of inflammatory pseudopolyps, suggesting potential clinical benefits in reducing unnecessary resections and streamlining procedures. However, further research with larger, diverse cohorts and the inclusion of more flat dysplastic lesions are required to confirm these findings.

## Supplemental Material

sj-docx-1-cmg-10.1177_26317745251363517 – Supplemental material for A pilot evaluation of the artificial intelligence system CAD-EYE to optically characterise lesions in inflammatory bowel disease surveillance Supplemental material, sj-docx-1-cmg-10.1177_26317745251363517 for A pilot evaluation of the artificial intelligence system CAD-EYE to optically characterise lesions in inflammatory bowel disease surveillance by Sherman Picardo, Shankar Menon, Kenji So, Kannan Venugopal, Wendy Cheng and Krish Ragunath in Therapeutic Advances in Gastrointestinal Endoscopy

## Declarations

**Ethics approval and consent to participate:** This is a retrospective image-based study. Ethics approval was obtained through the Western Australia (WA) Health: Governance, Evidence, Knowledge, Outcomes (GEKO) system for assessment of audit and quality activities (Approval Number: QA50366).

**Availability of data and materials:** Deidentified data and materials can be requested directly by contacting the corresponding author.
